# Knowledge Mapping of Multicriteria Decision Analysis in Healthcare: A Bibliometric Analysis

**DOI:** 10.3389/fpubh.2022.895552

**Published:** 2022-06-09

**Authors:** Zeqi Dai, Simin Xu, Xue Wu, Ruixue Hu, Huimin Li, Haoqiang He, Jing Hu, Xing Liao

**Affiliations:** ^1^Center for Evidence-Based Chinese Medicine, Institute of Basic Research in Clinical Medicine, China Academy of Chinese Medical Sciences, Beijing, China; ^2^Department of Cardiology, Guang'anmen Hospital, China Academy of Chinese Medical Sciences, Beijing, China; ^3^Evidence-Based Medicine Center, Beijing Hospital of Traditional Chinese Medicine, Beijing Institute of Traditional Chinese Medicine, Capital Medical University, Beijing, China

**Keywords:** bibliometric analysis, healthcare, multicriteria decision analysis, R-bibliometrix, VOSviewer, CiteSpace

## Abstract

**Objective:**

Multicriteria decision analysis (MCDA) is a useful tool in complex decision-making situations, and has been used in medical fields to evaluate treatment options and drug selection. This study aims to provide valuable insights into MCDA in healthcare through examining the research focus of existing studies, major fields, major applications, most productive authors and countries, and most common journals in the domain.

**Methods:**

A bibliometric analysis was conducted on the publication related to MCDA in healthcare from the Web of Science Core Collection (WoSCC) database on 14 July 2021. Three bibliometric software (VOSviewer, R-bibliometrix, and CiteSpace) were used to conduct the analysis including years, countries, institutes, authors, journals, co-citation references, and keywords.

**Results:**

A total of 410 publications were identified with an average yearly growth rate of 32% (1999–2021), from 196 academic journals with 23,637 co-citation references by 871 institutions from 70 countries/regions. The United States was the most productive country (*n* = 80). Universiti Pendidikan Sultan Idris (*n* = 16), Université de Montréal (*n* = 13), and Syreon Research Institute (*n* = 12) were the top productive institutions. A A Zaidan, Mireille Goetghebeur and Zoltan Kalo were the biggest nodes in every cluster of authors' networks. The top journals in terms of the number of articles (*n* = 17) and citations (*n* = 1,673) were *Value in Health* and *Journal of Medical Systems*, respectively. The extant literature has focused on four aspects, including the analytic hierarchy process (AHP), decision-making, health technology assessment, and healthcare waste management. COVID-19 and fuzzy TOPSIS received careful attention from MCDA applications recently. MCDA in big data, telemedicine, TOPSIS, and fuzzy AHP is well-developed and an important theme, which may be the trend in future research.

**Conclusion:**

This study uncovers a holistic picture of the performance of MCDA-related literature published in healthcare. MCDA has a broad application on different topics and would be helpful for practitioners, researchers, and decision-makers working in healthcare to advance the wheel of medical complex decision-making. It can be argued that the door is still open for improving the role of MCDA in healthcare, whether in its methodology (e.g., fuzzy TOPSIS) or application (e.g., telemedicine).

## Introduction

Since the emergence of multicriteria decision analysis (MCDA) in the 1970s (1), it has been widely used in many non-health sectors (2–6), such as energy, environment, military, management, architecture, and local and central government. MCDA is a sub-discipline of operations research with foundations in economics, management, mathematics, and psychology. MCDA is a process that integrates objective measurement with value judgment while also attempting to manage subjectivity (7). When facing complex decision-making situations in which multiple and conflicting criteria, objectives, or attributes are combined or aggregated, MCDA is a decision aid that helps stakeholders summarize complex value trade-offs in a way that is consistent and transparent, thus leading to fairer decision-making (7, 8). In the last decade, MCDA has been increasingly used as a transparent, participatory framework for supporting decision-making and policy setting in healthcare (9, 10). Decision-making in healthcare is inherently complex, as it usually involves confronting trade-offs between multiple objectives, requires the involvement of many stakeholders, and is often a constrained resource. This is particularly common in the field of health technology assessment (HTA). Decision-makers use explicit methods to determine the value of health technology at different points in its lifecycle (11). Evidence and Value:Impact on DEcisionMaking (EVIDEM) framework was developed by combining standardized HTA report with MCDA to promote transparent and efficient healthcare decision-making through systematic assessment and dissemination of the evidence and values on which decisions are based (12, 13).

MCDA can quantify benefits, risks, and uncertainties arising in decision-making, by considering an explicit set of criteria and their relative importance under a fully transparent process, while incorporating a wide range of stakeholder views to express a more societal perspective (14). MCDA process broadly involves problem structuring (i.e., selection of participants, alternatives, and criteria); modeling (i.e., weighting, scoring, and aggregation); and decision-making (i.e., interpretation of results and decision-making) (7). MCDA has been used in healthcare in different contexts, ranging from the marketing authorization stage, coverage decisions, the decision while prescribing, to other types of policy questions (14).

There have been several literature reviews that assessed different MCDA methods applied in healthcare decision-making under specific healthcare contexts. For instance, one systematic review in 2020 analyzed the use of multicriteria software in health priority settings and found that only a few studies used MCDA software in healthcare decision-making (15). One systematic review in 2015 showed that MCDA has been applied to a broad range of areas in healthcare, with the use of a variety of methodological approaches (16). Another systematic review in 2015 reported the applications of MCDA methods in decisions addressing the trade-off between costs and benefits at specific phases of medical innovation (17). One scoping review in 2019 assessed 70 case studies about the application of MCDA from three aspects: type of health services, type of interventions, and healthcare area (18). One review in 2014 assessed the value of healthcare interventions using MCDA, including pharmaceuticals, public health interventions, screening, surgical interventions, and devices (19). Recently, an extensive narrative review described the performance of fuzzy MCDA in the emergency system in the coronavirus disease 2019 (COVID-19) pandemic (20). It highlighted the importance of the fuzzy MCDA method as a beneficial tool for healthcare workers and first responders' emergency professionals to face this pandemic. Almost all of these reviews concluded and highlighted the need for guidance on the application of MCDA. Thus, it is imperative to provide an overall quantitative and longitudinal perspective of the works on this topic over time.

Bibliometrics refers to the quantitative analysis of all the knowledge carriers of a certain discipline using statistical and mathematical methods (21). It can be used not only to examine the history of scientific research in a specific field but also to identify potential future research directions and collaboration opportunities. In this study, scientometric visualization softwares—VOSviewer, R—bibliometrix, and CiteSpace—were used as a text mining and visualization tool for bibliometric analysis (22–24). Two previous bibliometric literature analyses of MCDA were performed covering the period from 1960 to 2011 (9) and 1980 to 2013 (16), respectively. Therefore, to help realize the current evidence landscape of global MCDA studies, we conducted a new bibliometric analysis of MCDA research to characterize global collaboration patterns, and map the developmental trends of MCDA over the past years.

The purpose of this study is to (1) analyze the distribution of publication outputs, countries, institutes, authors, journals, keywords, and references on MCDA research; (2) identify the cooperation of countries and institutes; (3) and explore the research trend and existing hotspots, which will help readers learn more about MCDA in healthcare, and the justification and significance of this study's analysis are obtained from two research topics: issues in the literature review on MCDA and the future trends. The following two suggested research questions (RQ) will help the study accomplish its aims.

RQ1.What changes have occurred in the literature on MCDA (e.g., the most influential research, important references with the most impact on the studies, the journals on this topic, and the change in the number of publications over time)?
RQ2.What are the most important topics and problems discussed in the scholarly literature on MCDA?

## Methods

### Data Source

In view of the special requirements of bibliometric software for data structure and content, the data used for analysis were collected from the following Web of Science Core Collection (WoSCC) indexes: the “Science Citation Index-Expanded (SCI-EXPANDED) (1985–2021)”, “Social Sciences Citation Index (SSCI) (1985–2021)”, “Emerging Sources Citation Index (ESCI) (2015–2021)”, and “Arts & Humanities Citation Index (A&HCI) (1985–2021)”, performed from 1 January 1985 to 14 July 2021. The searching terms were as follows: TS = (“multi-criteria decision analysis” OR “multiple criteria decision analysis” OR “multicriteria decision analysis” OR “multi-criteria decision making” OR “multicriteria decision making” OR “multiple criteria decision making” OR “multi-attribute decision analysis” OR “multiple attribute decision analysis” OR “multiattribute decision making” OR MCDA OR MCDM) AND TS = (“health care” OR healthcare OR health-care). We restricted our search results to papers published in English only. And the literature types are limited to Article and Review.

Finally, literature records, including titles, abstracts, and cited references, downloaded as plain text, formed the local database for subsequent analysis with VOSviewer, CiteSpace, and R-bibliometrix. The records were preprocessed with the deduplication function of CiteSpace.

### Study Selection

To avoid the bias caused by frequent database updates, all literature was retrieved and downloaded on the same day (14 July 2021). The title and abstract were read by two authors independently. All the research marked as potentially relevant by either author was advanced to the full-text review stage. Two authors read each selected full-text article separately to determine which research would be included based on the pre-defined selection criteria. Any discrepancies were resolved by the third author.

### Bibliometric Analysis and Visualization

In this study, Microsoft Office Excel 2016 was used to manage the data, and analyze the publication trend with linear regression. Publication data including publication counts, countries, institutes, authors, journals, references, and keywords were extracted from the WoSCC search results. The impact factor (IF) of the academic journals was collected from the 2020 Journal Citation Reports (JCR) (Clarivate Analytics, Philadelphia, United States). VOSviewer (1.6.17), a network analysis software tool used to analyze bibliometric data, was used to identify productive countries/regions, institutions, authors, and co-occurrence keywords. In VOSviewer network maps, different nodes indicate components, while the size of the nodes reflects the number of publications or co-occurrence frequencies. The links between nodes represent the co-occurrence relationships, and the size of the links indicates the co-occurrence frequencies between nodes. The VOSviewer settings were as follows: counting method (full counting), while thresholds (T) of items (countries/regions, institutions, authors, and keywords) with more than five publications were adopted. The bibliometrix package in R (4.1.1) was utilized to analyze the research hotspots and trends with word dynamics, trend topic, and thematic map. CiteSpace software (5.7.R2) was utilized for visual exploration of the distribution of co-citation references with the timezone diagram. The visual network created consists of nodes and lines. The nodes in the timezone diagram indicate the co-citation references. The size of the nodes reflects the number of citation frequencies. The colors of the node and line represent different years. The X-axis represents the time. The parameters of CiteSpace were as follows: time (1999–2021), years per slice (1), links (strength: cosine, scope: within slices), and selection criteria (Top N Per slice = 10).

## Results

### Search Results

**Figure 1:**
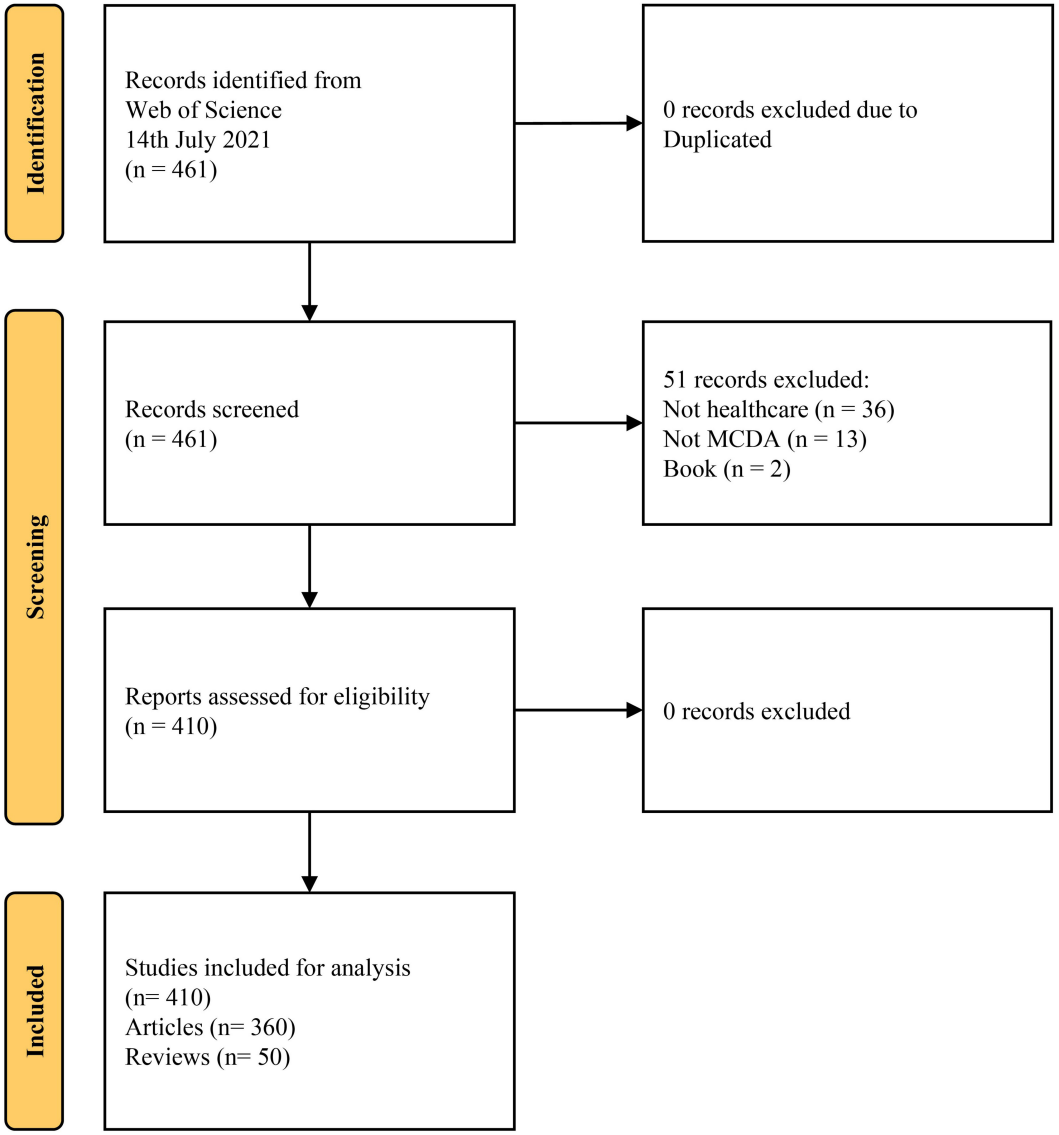
Flowchart of the study selection process.

A total of 461 articles in the WoSCC database were retrieved on 14 July 2021 (see Supplementary File 1). They were contained in four indexes, including SCI-Expanded (*n* = 353), SSCI (*n* = 253), ESCI (*n* = 58), and A&HCI (*n* = 1) (see Supplementary File 2). In total, the majority of the publications (*n* = 203) are indexed in both SCI-Expanded and SSCI Indexes, followed by SCI-Expanded alone (*n* = 150). Finally, 410 met the selection criteria and were used for the bibliometric analysis (Figure 1). The result of distribution by time indicates that the literature in this field was published between 1999 and 2021, including 360 articles and 50 reviews. They were published in 196 academic journals with 23,637 co-citation references by 871 institutions from 70 countries/regions around the world.

### Publication Outputs

**Figure 2:**
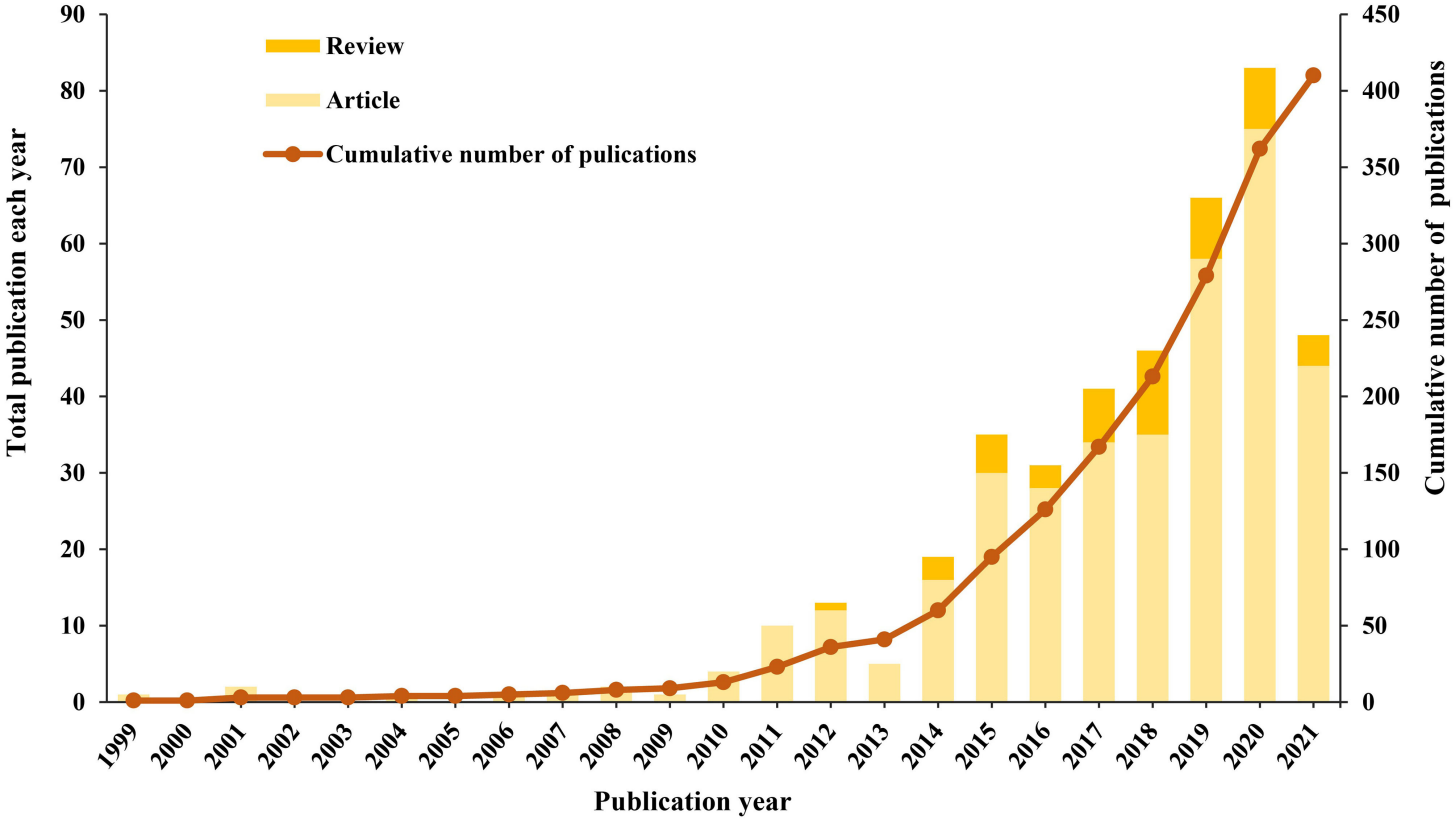
Annual trend chart of publications.

**Figure 3:**
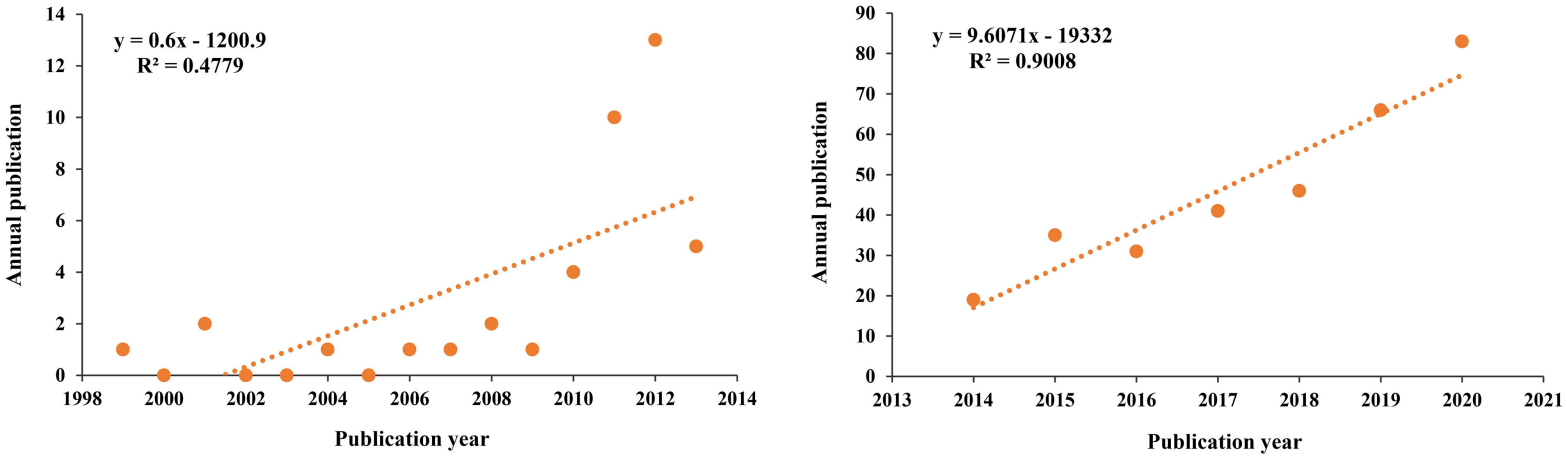
The curve fitting results of annual publications.

The annual growth trends of publications regarding MCDA are shown in Figure 2. There is a steady increase in publications by year, with a notably higher output in the last 5 years (2016–2021, 76.83%). The earliest published literature comes from 1999 and the publications increased at an average rate of 9.6 articles per year from 2016 to 2021. According to the characteristics of the publication outputs, a research period was divided into two periods (1999–2013, and 2014–2021) (Figure 3). The period from 1999 to 2013 was a slow growth period, with a small number of 41 articles published; 2014–2020 was a period of rapid growth, and the number of TP showed a significant ascending curve, which accounted for 90% of all the articles.

### Regions, Institutes, and Authors' Analysis

**Figure 4:**
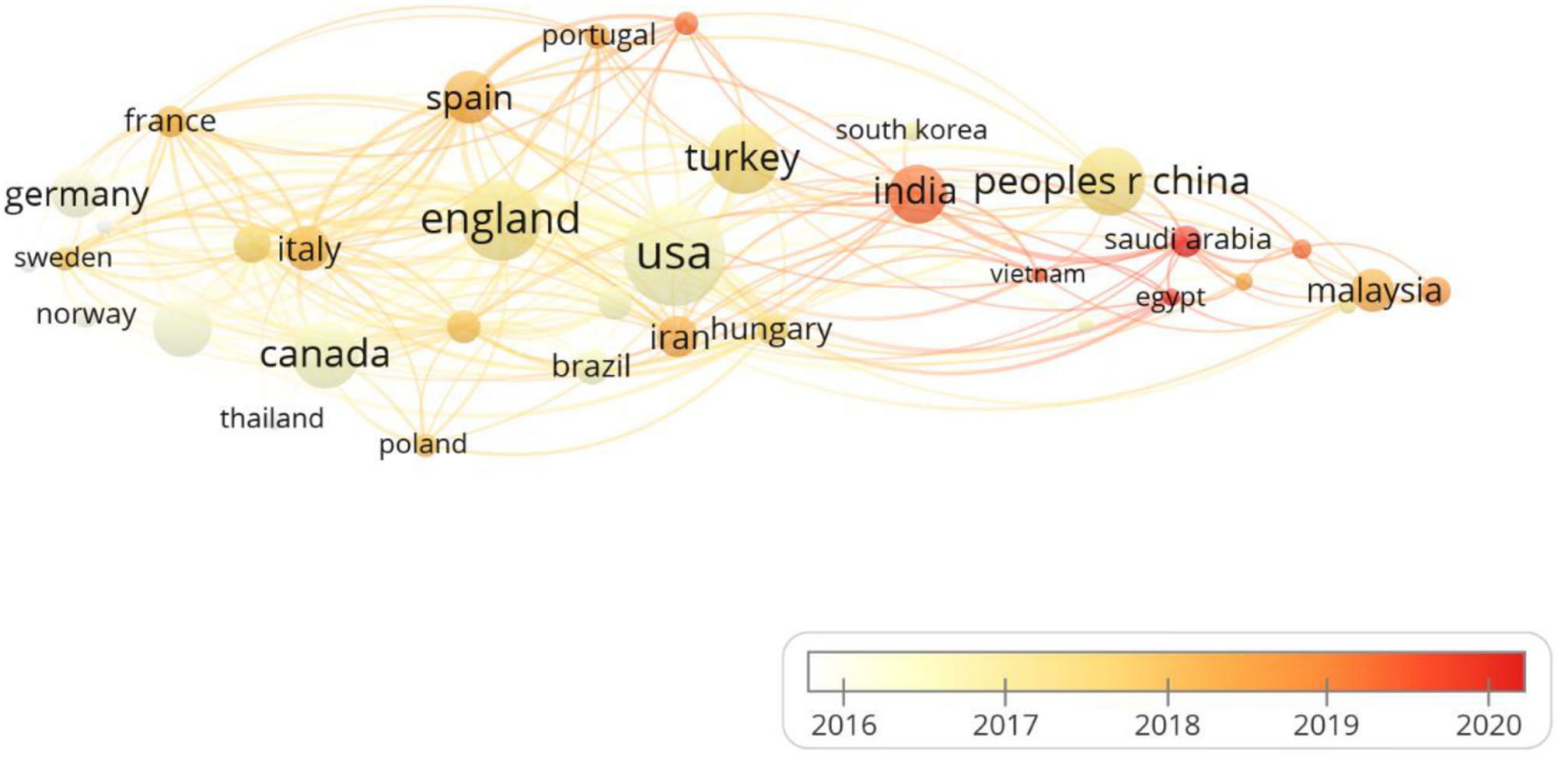
Collaboration network of countries/regions. The size of a single node represents the number of publications of the countries/regions. The color of the nodes represents the publication in different countries with the change of time (yellow: earlier, orange: later).

A total of 70 countries contributed to the publications on MCDA in healthcare, and 35 countries with more than five publications were analyzed (Figure 4). The top five ranked countries by publication count were United States (80), the United Kingdom (25), Turkey (26), China (27), and Canada (28).

**Figure 5:**
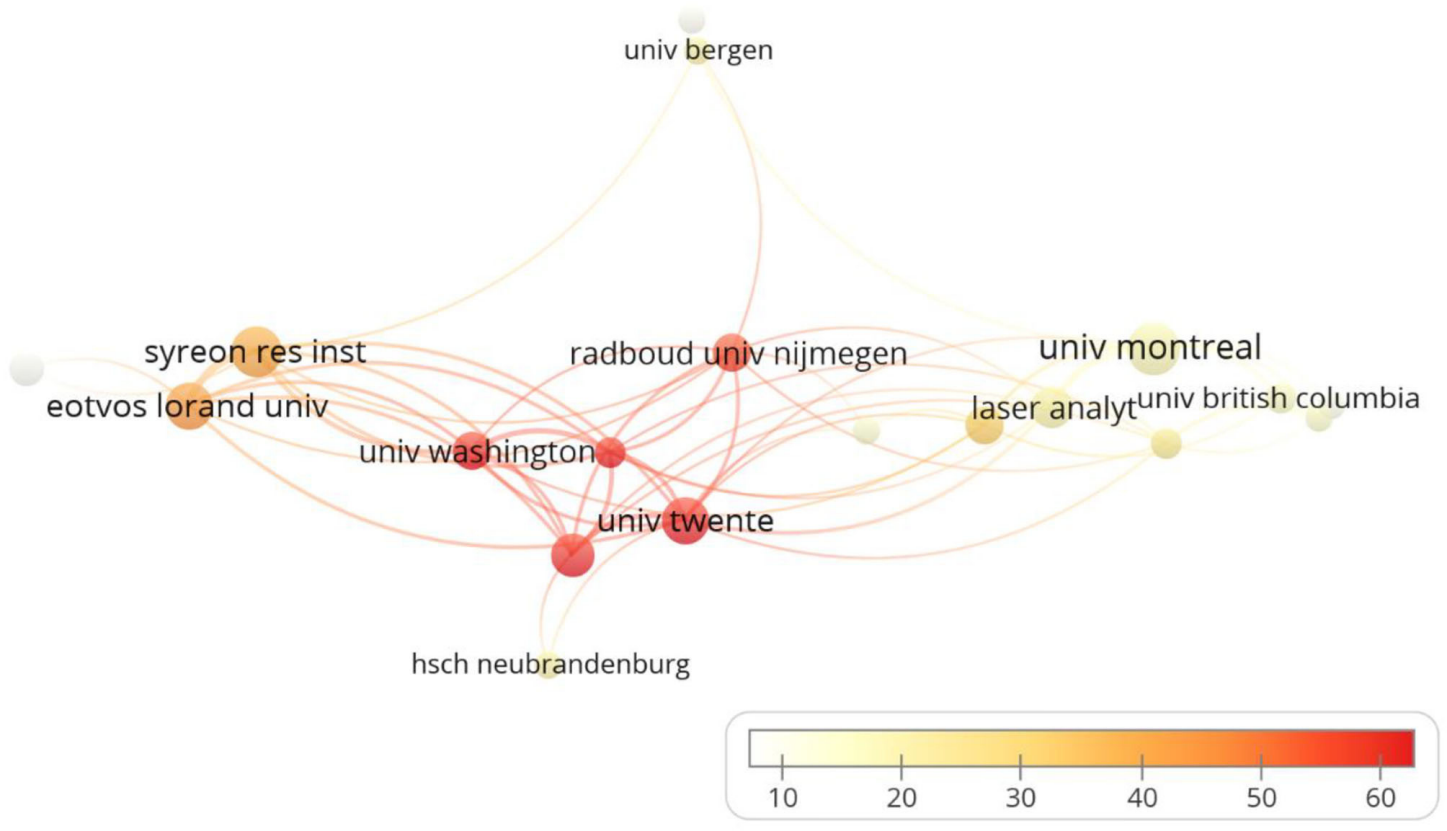
Collaboration network of institutions. The size of a single node represents the number of publications of the institutions. The color of the nodes represents the average citation in different institutions.

A total of 871 institutions published articles related to MCDA, out of which 34 institutions published more than five publications (Figure 5). The top leading institution by publication count was Universiti Pendidikan Sultan Idris (*n* = 16) in Malaysia, mainly published by the Department of Computing. Besides, Université de Montréal (*n* = 13) in Canada was the second, followed by Syreon Research Institute (*n* = 12) in Hungary, University of Twente (*n* = 11) in the Netherlands, and Eötvös Loránd University (*n* = 11) in Hungary. In terms of average citations (Citations/Documents) of articles, Office Health Economics (492/6), the United Kingdom; University of Washington (536/8), the United States; Tongji University (460/7), China; Shanghai University (382/6), China; and University of Twente (685/11), the Netherlands, have higher average citations.

**Figure 6:**
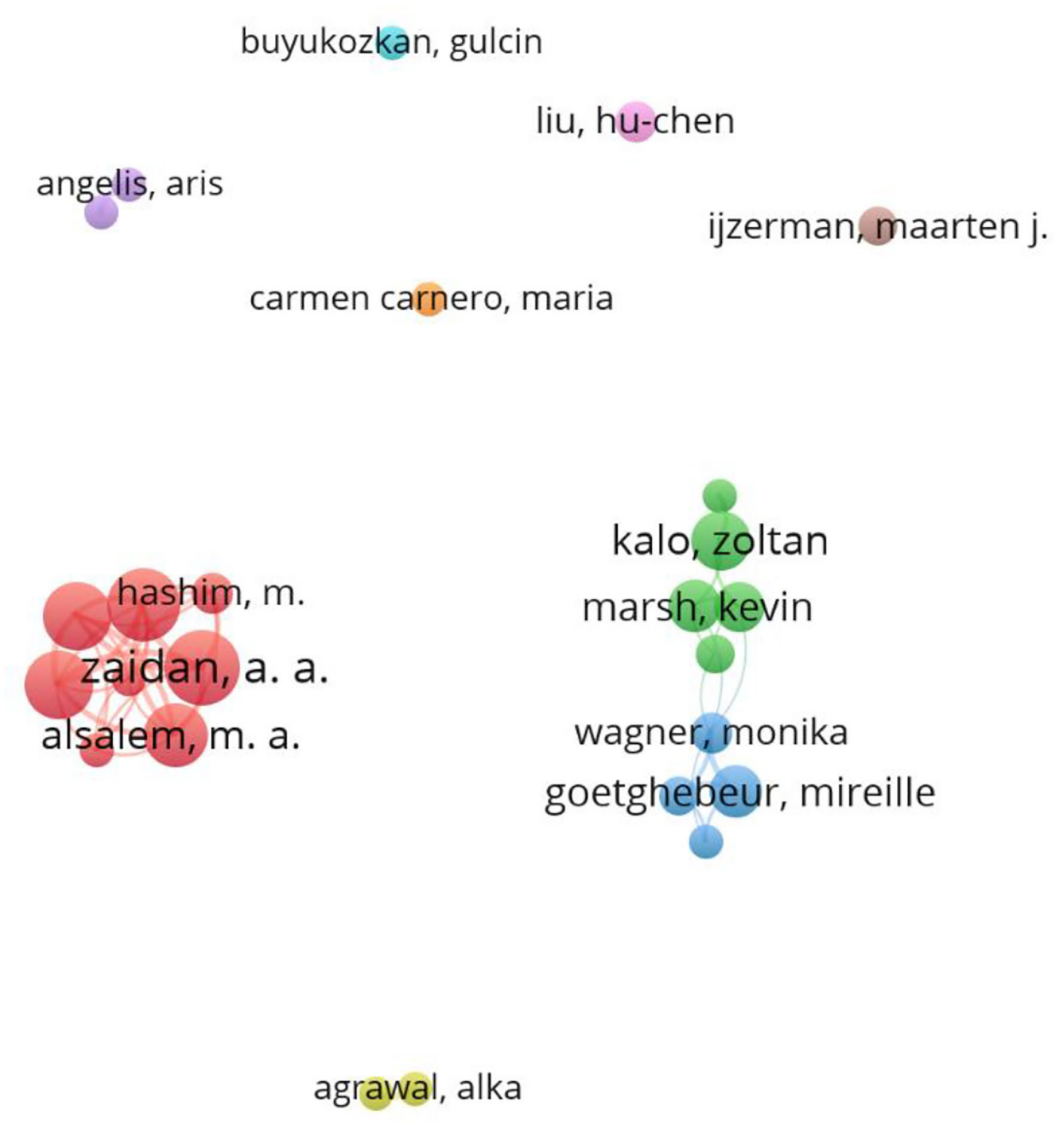
Collaboration network of authors. The size of a single node represents the number of publications of the author. Each color represents a different cluster.

A total of 1,475 authors published articles related to MCDA, and 25 authors with more than five publications were analyzed (Figure 6). The top 10 leading authors by publication count published 124 articles in total. A A Zaidan (*n* = 17) ranked first, followed by B B Zaidan (*n* = 16), O S Albahri (*n* = 15), A S Albahri (*n* = 15), M A Alsalem (*n* = 13), Zoltan Kalo (*n* = 12), Mireille Goetghebeur (*n* = 10), Kevin Marsh (*n* = 10), Rob Baltussen (*n* = 9), and M Hashim (*n* = 7). Figure 6 shows the network visualization between the coauthors of MCDA research. Three teams contributed to most publications. The biggest cluster mainly consisted of A A Zaidan, M A Alsalem, and M Hashim who are from Universiti Pendidikan Sultan Idris. The next cluster is mainly composed of Zoltan Kalo and Kevin Marsh, followed by the third cluster with Mireille Goetghebeur and Monika Wagner.

### Journal Analysis

A total of 410 publications were published in 196 academic journals and were cited 23,637 times. The 15 most productive journals and 15 most co-citation journals in the MCDA research are presented in Table 1. The most productive journal was the *Value in Health* (*n* = 17, IF2020 = 5.725, Q1), followed by *BMC Medical Informatics and Decision Making* (*n* = 13, IF2020 = 2.796, Q3), *Expert Review of Pharmacoeconomics & Outcomes Research* (*n* = 12, IF2020 = 2.217, Q3/Q4), *Journal of Medical Systems* (*n* = 11, IF2020 = 4.460, Q1/Q2), and *Cost Effectiveness and Resource Allocation* (*n* = 9, IF2020 = 2.532, Q3). The 15 most co-citation journals had 8,967 citations. *Journal of Medical Systems* had the most citations (*n* = 1673), followed by *Value in Health* (*n* = 942), *BMC Medical Informatics and Decision Making* (*n* = 746), *Expert Systems with Applications* (*n* = 632), and *Expert Review of Pharmacoeconomics & Outcomes Research* (*n* = 563).

**Table 1:** The top 15 most productive journals and most co-citation journals in MCDA research.

**Rank**	**Journal**	**Count**	**IF2020^#^**	Q^*^	**Journal**	**Co-citation Count**	**IF2020**	**Q**
1	Value in Health	17	5.725	Q1	Journal of Medical Systems	1,673	4.460	Q1, Q2
2	BMC Medical Informatics and Decision Making	13	2.796	Q3	Value in Health	942	5.725	Q1
3	Expert Review of Pharmacoeconomics and Outcomes Research	12	2.217	Q3, Q4	BMC Medical Informatics and Decision Making	746	2.796	Q3
4	Journal of Medical Systems	11	4.460	Q1, Q2	Expert Systems with Applications	632	6.954	Q1
5	Cost Effectiveness and Resource Allocation	9	2.532	Q3	Expert Review of Pharmacoeconomics and Outcomes Research	563	2.217	Q3, Q4
6	BMC Health Services Research	8	2.655	Q3	IEEE Access	549	3.367	Q2
7	Journal of Cleaner Production	8	9.297	Q1	Pharmacoeconomics	508	4.981	Q1
8	Pharmacoeconomics	8	4.981	Q1	Journal of Multi-criteria Decision Analysis	481	-	-
9	Expert Systems with Applications	7	6.954	Q1	International Journal of Environmental Research and Public Health	473	3.390	Q1, Q2
10	IEEE Access	7	3.367	Q2	Journal of Cleaner Production	467	9.297	Q1
11	International Journal of Environmental Research and Public Health	7	3.390	Q1, Q2	Frontiers in Public Health	462	3.709	Q1, Q2
12	Journal of Multi-criteria Decision Analysis	7	-	-	Cost Effectiveness and Resource Allocation	394	2.532	Q3
13	International Journal of Technology Assessment in Healthcare	6	2.188	Q3, Q4	BMC Health Services Research	381	2.655	Q3
14	Medical Decision Making	6	2.583	Q2, Q3	Health and Technology	364	-	-
15	Waste Management and Research	6	3.549	Q2, Q3	Health Expectations	332	3.377	Q1, Q2

# *IF, Impar Factor;*

* *Q, Quartile in Category*.

### Co-citation Analysis

**Figure 7:**
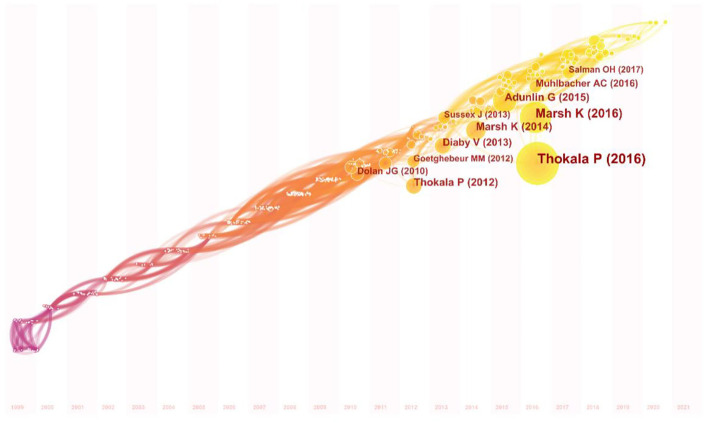
References' timezone network. The nodes indicate the co-citation references. The size of the nodes reflects the number of citation frequencies. The colors of the node and line represent different years. The X-axis represents the time.

A timezone graph is a view of representing knowledge evolution in time. All the nodes in the timezone graph are located in a two-dimensional coordinate with the horizontal axis of time. According to the time of the first cited, the nodes are set in different timezones, and their positions are upward with the time axis. The co-citation timezone graph can clearly show the number and relationship of co-citation references in different periods. The top 10 cited references were as follows, Thokala P 2016 (29) (*n* = 69), Marsh K 2016 (30) (*n* = 45), Adunlin G 2015 (16) (*n* = 27), Marsh K 2014 (19) (*n* = 26), Thokala P 2012 (31) (*n* = 24), Diaby V 2013 (9) (*n* = 23), Muhlbacher AC 2016 (32) (*n* = 18), Dolan JG 2010 (33) (*n* = 16), Sussex J 2013 (34) (*n* = 14), and Goetghebeur M 2012 (35) (*n* = 14) (see Figure 7).

### Keywords Analysis

**Figure 8:**
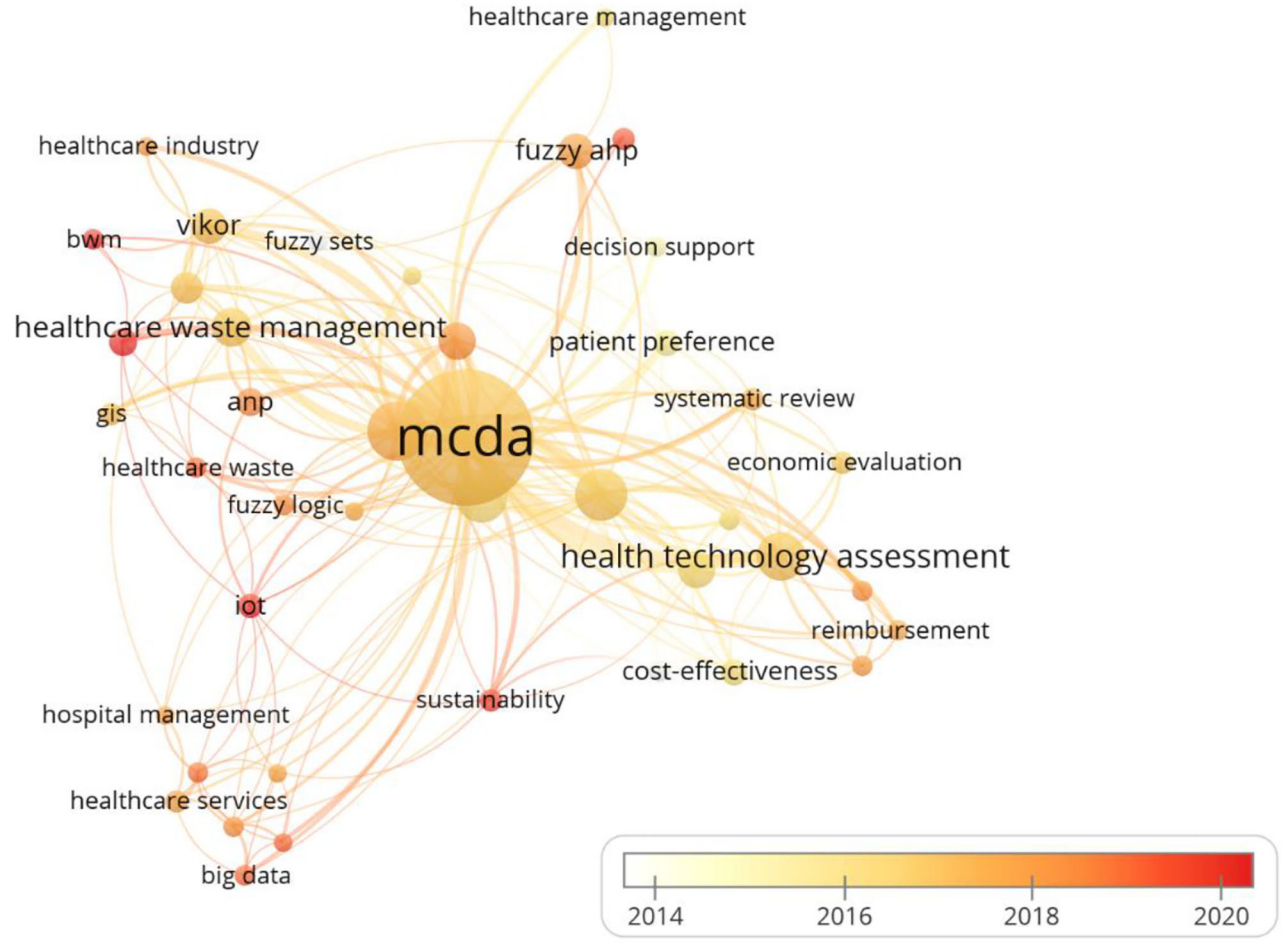
Co-occurrence network of keywords according to the average publication year. The size of a single node represents the number of publications of each keyword. The color of the nodes represents the emergence time of different keywords (yellow: earlier, orange: later).

An analysis of keywords used in articles on MCDA is shown in Figure 8. After merging the synonyms of keywords, such as “multiple criteria decision analysis” and “multi-criteria decision analysis” into “MCDA,” a total of 1,091 keywords were found. We analyzed 42 keywords that were identified as having occurred more than five times (Figure 8). The results showed that the top 10 keywords are “MCDA” (*n* = 224), “analytic hierarchy process (AHP)” (*n* = 46), “decision-making” (*n* = 35), “healthcare” (*n* = 34), “HTA” (*n* = 31), “healthcare waste management (HWM)” (*n* = 20), “priority setting” (*n* = 19), “technique for order preference by similarity to an ideal solution (TOPSIS)” (*n* = 18), “fuzzy AHP” (*n* = 16), and “Vise Kriterijumska Optimizacija I Kompromisno Resenje (VIKOR)” (*n* = 16). The colors in the overlay visualization indicated the average publication year of the identified keywords. The majority of keywords were published after 2016, with yellow or orange color.

To further study the research hotspot tendency, we conducted the word dynamics, trend topic, and thematic map analysis by R-bibliometrix.

**Figure 9:**
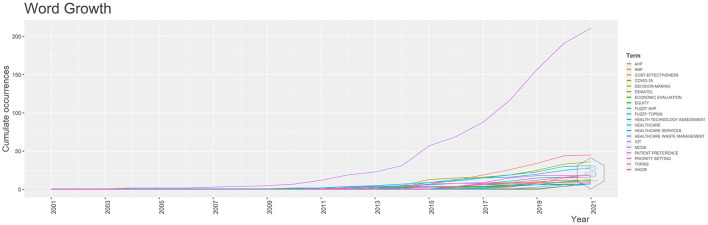
The cumulative growth keywords (top 20).

Analyzing the annual growth of keywords, the top 20 keywords, such as “MCDA”, “AHP”, “decision-making”, “HTA”, “healthcare”, and “priority setting”, are shown in Figure 9. Among them, “MCDA” showed the J-shaped curves, with sustained growth from a continuous growth trend from 2001 to 2021. The growth trend of four keywords (“AHP”, “decision-making”, “HTA”, and “healthcare”) is relatively stable compared with “MCDA”. The most recent “COVID-19” grew rapidly.

**Figure 10:**
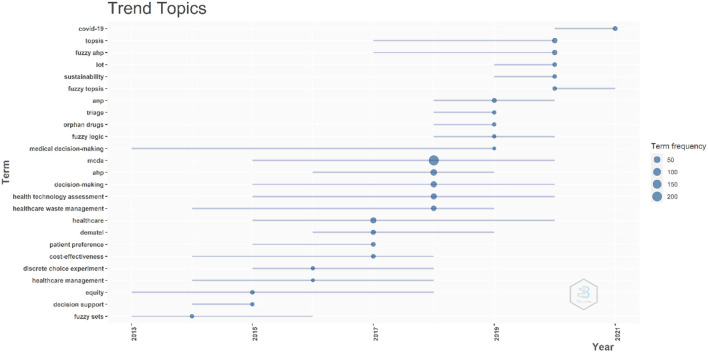
Trend topics. The X-axis represents the year, while the Y-axis is the cumulate occurrences of the keywords.

The trend topic analysis is an important mapping tool that helps to portray the seed of trend integration rooted in the previous stream. The buzz topics in MCDA research are shown in Figure 10. The following topics have been identified when examining the authors' keywords and maintaining a minimum five-word frequency and maximum of ten words per year. Except for the words “MCDA” and “healthcare” that we searched for, keywords such as “medical decision-making”, “decision-making”, “HTA”, “HWM”, and “equity” had the longest duration of 6 and 5 years, respectively. Here, the notable topics are “HTA” and “HWM”, which have both a higher frequency and longer duration. Very recently, in 2020 and 2021, “COVID-19” and “TOPSIS” have also been examined.

**Figure 11:**
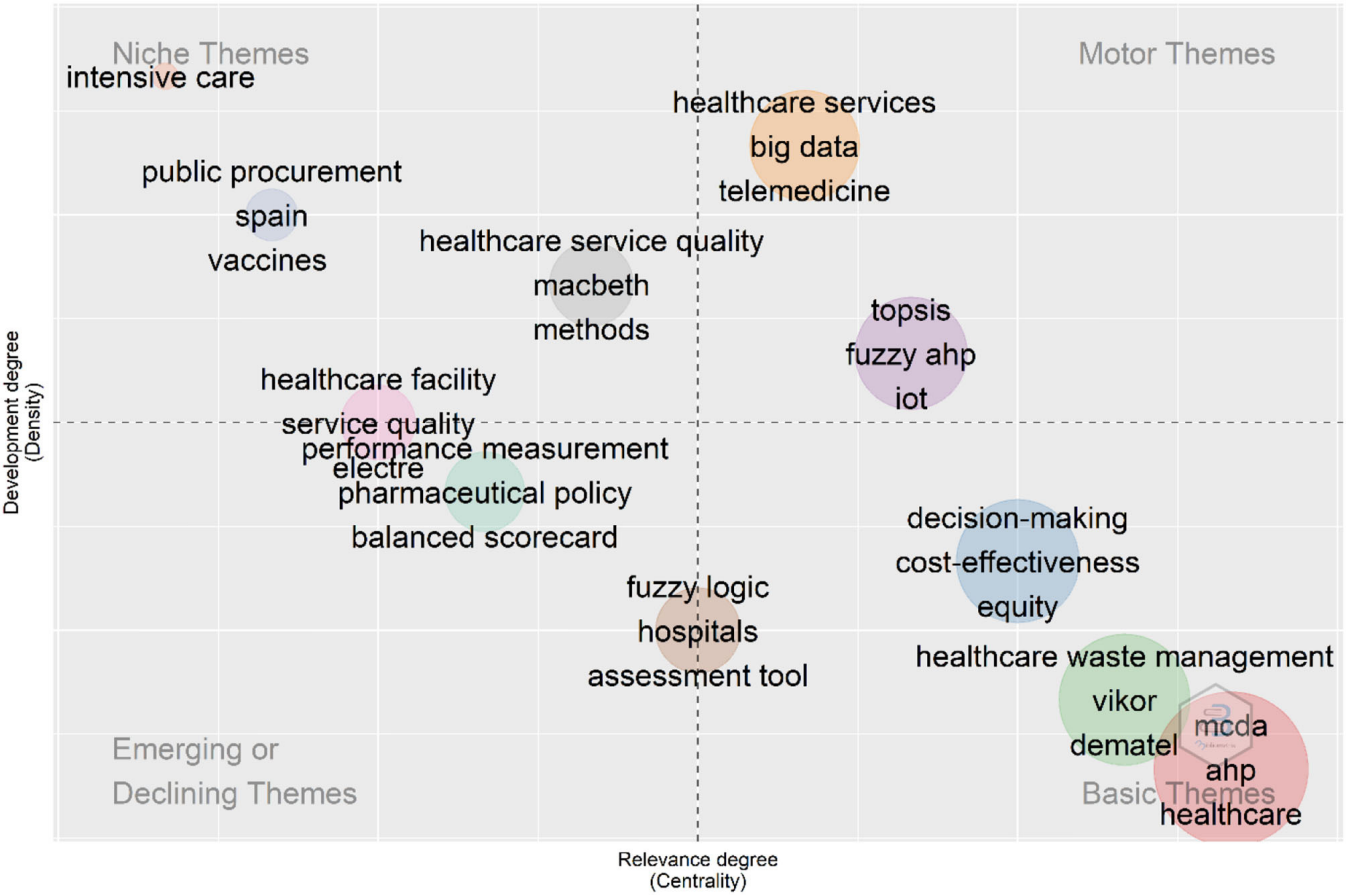
Thematic map showing clusters and the keywords from 1999 to 2021 identified by the co-occurrence network. The bubble size is proportional to the occurrences of the words in the cluster. The X-axis represents the centrality (i.e., the degree of interaction of a network cluster in comparison with other clusters) and gives information about the importance of a theme. The Y-axis symbolizes the density (i.e., measures the internal strength of a cluster network, and it can be assumed as a measure of the theme's development).

Finally, we conducted the keywords thematic map, which allows visualization of four different typologies of themes based on two dimensions namely- density- that is the strength of internal ties among all the keywords that are used to describe the research theme and centrality–that is the strength of external ties to other themes by exploiting the authors' keyword field as shown in Figure 11. For the creation of a thematic map, a total of 300 keywords were examined where a minimum cluster frequency is 5 and the number of labels for each cluster is 2. The motor theme, the upper right quadrant, which is characterized by a high density and centrality shows probably the well-developed and important themes for the structuring of the MCDA research field, classifying them into two clusters. Cluster one includes “healthcare services”, “big data”, and “telemedicine”, and the second one includes “TOPSIS” and “fuzzy AHP”. In the upper-left quadrant (niche themes), it is possible to find the themes “healthcare service quality”, “public procurement”, and “healthcare facility” as major keywords. The cluster in the third quadrant (emerging or declining themes) was characterized by low centrality and density, which means that it was weakly developed and marginal, including “performance measurement” and “pharmaceutical policy” as the principal. The fourth quadrant (basic themes) contained “decision-making”, “cost-effectiveness”, “HWM”, “VIKOR”, “MCDA”, and “AHP” as the most common keywords. They concern general topics that are transversal to different research areas of the field.

## Discussion

Within the aim of the study, the current bibliometric analysis provided information about the structure of MCDA publications in the field of healthcare in various categories, which helped researchers recognize publication activities in relation to citations, journals, authors, etc. Since 2014, the number of scholarly MCDA publications has increased steadily. The earliest record was published in 1999 (36) and reported the process of health technology procurement in a University Hospital in Rio de Janeiro in Brazil.

The countries where the research was conducted can be summarized by bibliometric analysis. Several bibliometric analysis studies in different fields (37–39) showed that developed countries have more publications, and low-income countries need not only to promote their research but also to establish research collaboration with developed countries. In our study, North America (e.g., the United States, Canada), Europe (e.g., the United Kingdom, the Netherlands, Spain, Germany, Italy), and Asia (e.g., Turkey, China) have published a significant number of MCDA publications, which is similar to the findings of a systematic review (40). Among them, the United States which is the most productive country, has conducted MCDA research since 2001, followed by Turkey since 2006 and Canada since 2008. In the past decade, the United Kingdom, China, the Netherlands, India, and Spain have also started MCDA research. Both developed and developing countries have made certain contributions to MCDA research. Although Malaysia, Hungary, and other countries did not rank in the top 10 in terms of the publication number, however, for institutions, Universiti Pendidikan Sultan Idris in Malaysia and Syreon Research Institute in Hungary ranked first and third separately.

In terms of authors, there are three large cooperation teams in the current research field. The first is the core team formed by A A Zaidan, B B Zaidan, O S Albahri, A S Albahri, and M A Alsalem. These authors are mainly from Universiti Pendidikan Sultan Idris (UPSI), and have published 13 articles, following the research of MCDA and telemedicine closely, such as real-time remote-health monitoring systems (41), the new m-health hospital selection framework (42). However, this collaborative author network is separate from other networks, indicating that it rarely cooperates with other authors or teams. Second, Zoltan Kalo, Kevin Marsh, and Rob Baltussen cooperated closely and participated in two reports on MCDA used in healthcare decision-making by the task forces of the International Society for Pharmacoeconomics and Outcomes Research (ISPOR) (29, 30). Task forces have developed ISPOR's Good Practices Reports, which are highly cited expert consensus guidance recommendations that set international standards for outcomes research and its use in healthcare decision-making (43). Furthermore, three authors also cooperated with other authors extensively. Zoltan Kalo focused on the research of health economics, such as the weighting methods used in MCDA frameworks in healthcare, potential criteria for frameworks to support the evaluation of innovative medicines in the upper-middle-income countries, and the potential impact of MCDA on pricing and reimbursement process of orphan drugs in Poland. Rob Baltussen has concerns for HIV/AIDS and HTA, such as priority setting of HIV/AIDS interventions, MCDA to support HTA agencies, and value assessment frameworks for HTA agencies. Kevin Marsh emphasizes more on preference information and decision analysis, such as lessons from MCDA for quantifying patient preferences and prioritizing investments in new vaccines against epidemic infectious diseases. Third, Mireille Goetghebeur, Monika Wagner, and Hanane Khoury from LASER Analytica, also worked closely and published four articles together (28, 35, 44, 45) on EVIDEM and HTA. First, they piloted a proof-of-concept evaluation using the EVIDEM framework to appraise 10 medicines covering six therapeutic areas. Then they used tramadol for chronic non-cancer pain as a case study, and the application of rare diseases has also been studied in recent years. In terms of the journal, both the most co-cited journals and productive journals played important roles in the MCDA research. Top co-cited journals could be used as reference sources while writing manuscripts and the top productive journals could be taken into consideration while submitting manuscripts (46).

A Co-citation network represents how frequently two publications are cited together by other publications; therefore, it can be regarded as a knowledge base in a special field (27). In this study, the top 10 co-citation references were selected to identify the knowledge base of MCDA. The two most-cited studies, with 69 and 45 citations separately, are the reports of ISPOR task forces on MCDA for healthcare decision-making (29, 30). Report 1 introduces MCDA, including definition, type, key steps, and application. Report 2 provides more in-depth practice guidance on the implementation of MCDA, including a checklist to guide the design and reporting. These two reports give fundamental advice on how to use MCDA best to support healthcare decision-making. The 3rd to 8th co-citation references are six reviews, involving literature review, systematic review, and bibliometrics analysis, of which five studies reviewed the research of MCDA in healthcare (9, 16, 19, 32, 33), and one study reviewed the application of MCDA in the field of HTA (31). The last two studies are both the application of MCDA. The 9th most-cited study piloted the use of MCDA to establish and apply a framework of weighted attributes to value orphan medicinal products (34), and found that the MCDA approach for rare disease treatment value assessment has the merit of ensuring shared understanding of the elements of value as well as a clear articulation of trade-offs between those elements. The 10th most-cited study (35) combined HTA with MCDA, and a pan-Canadian group of policy and clinical decision-makers and researchers appraising 10 medicines covering six therapeutic areas. This proof-of-concept study demonstrated the usefulness of incorporating MCDA in HTA to support a transparent and systematic appraisal of healthcare interventions. Generally, we found that the top 10 co-citations mainly discussed the definition, steps, approaches, application, and impact, all of which were the foundations of MCDA research.

Based on keywords co-occurrence, word dynamics, trend topic, and thematic map, the current potential research hotspots and trends were obtained. In the past decades, researchers of MCDA in healthcare have rapidly increased their attention to applied categories such as “HTA”, “HWM”, and “AHP”. At present, keywords such as “COVID-19” and “TOPSIS” have also attracted the attention of researchers, which may represent a trend in this field.

Many healthcare policy decision-makers have been studying the application of MCDA to support HTA decisions (32). HTA is a multidisciplinary process that uses explicit methods to determine the value of health technology at different points in its lifecycle (47). MCDA has the potential to enhance and complement the HTA decision-making process (26), and its application in HTA has received widespread attention. For instance, the workshop in Canada discusses opportunities and concerns with reference to the implementation of MCDA in Canada (26). Some researchers have also explored the application of MCDA in the assessment and appraisal of orphan drugs for HTA (48). The National Institute for Health and Clinical Excellence (NICE) is also considering MCDA as one of the ways to implement its new value-based pricing scheme (31).

HWM is also one of the potential hotspots of this research. Healthcare waste today poses grave challenges to hospitals and medical institutions, especially in developing countries where medical waste is very often combined with municipal waste, threatening the health and safety of the handling staff, the general public, and the environment (49). When planning a country's HWM system, decision-makers should consider a variety of parameters, including technical, economic, social, and political factors (50). At this point, MCDA will help decision-makers to solve this complex problem. For example, HWM research has been conducted in Myanmar, India, Istanbul, and other countries. Some researchers used VIKOR to find out the priority of healthcare waste disposal plans during COVID-19 (51).

Understanding the MCDA technologies is crucial. AHP as one of the hot MCDA technologies appears in the research. AHP aims to solve multifactorial and multidimensional problems. It decomposes a complex decision-making problem into different levels. And then, in the pairwise comparison, the weight of each criterion and alternative is judged, and the Eigenvector method is used to calculate the priority (52). AHP was developed by Saaty in the late 1970s (52, 53). Since 1989, it has been accepted slowly as a method of MCDA in healthcare (52). TOPSIS, as one of the classical MCDA technologies, had high frequency in the recent 2 years. TOPSIS is based on the idea that the chosen alternative should have the shortest distance from the positive ideal solution, and on the other side, the farthest distance from the negative ideal solution (54), ranking its advantages and disadvantages based on idealized goals after determining the closeness of its limited evaluation objects. Besides, VIKOR, Decision-making Trial and Evaluation Laboratory (DEMATEL), Evidence and Value: Impact on DEcision-Making (EVIDEM), and MULTIMOORA are also widely used in MCDA (25, 55–57). According to the different research questions, there are preferences in the choice of technologies (51).

It is universally known that COVID-19 broke out at the end of 2019 and remains a threat to global health. As one of the most recent research hotspots, the application of MCDA to COVID-19 mainly involves the following aspects: healthcare waste management (51, 58), prioritization of hospital admission of patients (59), the assessment of intervention and care (60, 61), selection of isolation hospital location (62), and evaluation of a national healthcare system (63). These studies exert impact on both decision-making and management of COVID-19, and this impact will likely continue in the next few years. This information will provide directions for research on other pandemics.

A thematic map can analyze which themes are likely to have long-term development in the future. The keywords, which appear in the motor themes, are both important and well-developed. It is evident that the motor theme now discusses two main perspectives. The cluster one included “healthcare services”, “big data”, and “telemedicine” as the main keywords, followed by “triage”, “hospital management”, “mobile health (m-health)”, and “prioritization”. Big data refer to data that are so large, fast, or complex that they is difficult or impossible to process using traditional methods. Big data are receiving increasing attention in biomedicine and healthcare. In the era of big data, telemedicine and m-health are increasingly used in the modern healthcare systems. Patients, providers, and payers can benefit from the emergence of telemedicine, in terms of the geographical location, costs, emergency treatment, child care/elder care challenges, and so on (64). During COVID-19, telemedicine can attempt to reduce the spread between patients, families, and clinicians (65). However, telemedicine faces challenges in triage and prioritization. MCDA may be one of the solutions for dealing with these challenges. After incorporating the MCDA, people may consider the multiple criteria simultaneously and assign appropriate weight to each criterion, and score patients according to their urgency (66), which optimizes the process of triage and prioritization. The second cluster in motor themes mainly includes “TOPSIS” and “fuzzy AHP”, as well as “internet of things (IOT)”, “medical services”, and other keywords. Based on this result and the previous analysis, MCDA technology has always been one of the research hotspots and the key technologies supporting MCDA research.

Our study analyzed the global trends and application status of MCDA research over 23 years in healthcare from the WoSCC database. It provided insights into scientific research, which will assist in generating evidence-based descriptions, comparisons, and visualizations of research output in MCDA by employing three bibliometric softwares. Nevertheless, our analyses have some limitations. First, the searches were only conducted in the WoSCC database and only in the English language. This can lead to selection bias due to the omission of some studies. Second, as this is an emerging and developing research field and the time we searched was up to July 2021, we might have underestimated the contribution to different analyses of the recently published studies. Third, we only analyzed the information such as authors, keywords, and citations of the included studies from the perspective of bibliometrics, and did not analyze the specific content of the studies. Therefore, the analysis may not provide a better overview of the MCDA-published literature.

## Conclusion

The total number of publications shows exponential growth in the past decades. The author teams are mainly from UPSI, LASER Analytica, Syreon Research Institute, and Evidera. The hotspots mainly included AHP, HTA, and HWM, and concentrated on COVID-19 and fuzzy TOPSIS, recently. Furthermore, big data, telemedicine, TOPSIS, and fuzzy AHP, which are well-developed and important themes, might be the trends in future research. The wider use of MCDA, in either methodology or application, will help promote complex decision-making.

## Author Contributions

ZD: conceptualization, methodology, software, investigation, formal analysis, visualization, and writing—original draft. SX: investigation, visualization, and writing—original draft. XW, RH, and HL: investigation. HH: formal analysis and visualization. JH: writing—review and editing. XL: conceptualization, supervision, and writing—review and editing. All authors contributed to the article and approved the submitted version.

## Funding

This study was supported by the National Natural Science Foundation of China (No. 82174239), CACMS Innovation Fund (No. CI2021A00701-3), the Fundamental Research Funds for the Central Public Welfare Research Institutes (No. ZZ13-YQ-075), China Center for Evidence Based Traditional Chinese Medicine, CCEBTCM (No. 2020YJSZX-2), and the 14th Basic Scientific Research Project of CACMS (Z0718, Z0724, Z0754).

## Conflict of Interest

The authors declare that the research was conducted in the absence of any commercial or financial relationships that could be construed as a potential conflict of interest.

## Publisher's Note

All claims expressed in this article are solely those of the authors and do not necessarily represent those of their affiliated organizations, or those of the publisher, the editors and the reviewers. Any product that may be evaluated in this article, or claim that may be made by its manufacturer, is not guaranteed or endorsed by the publisher.
